# Acute high-grade acromioclavicular joint dislocation patients treated with titanium cable insertion under a homemade guider

**DOI:** 10.1186/s13018-021-02442-1

**Published:** 2021-04-30

**Authors:** Jun Wang, Yongfeng Cui, Yuhang Zhang, Hang Yin

**Affiliations:** Department of Orthopedic, Xiaoshan First People’s Hospital, Xiaoshan Affiliated Hospital of Wenzhou Medical University, No. 199 Shixin Road, Hangzhou, 311200 Zhejiang Province China

**Keywords:** Acromioclavicular, Dislocation, Guide, Technique, Rockwood

## Abstract

**Backgrounds:**

To describe a new technique for implanting a double-bundle titanium cable to treat acromioclavicular (AC) joint dislocation via the new guider, and evaluate clinic outcomes.

**Methods:**

A retrospective study of patients treated for acute high-grade acromioclavicular joint dislocation from June 2016 to January 2020 in our trauma center, twenty patients with AC joint dislocation were managed with double-bundle titanium cable. It includes the following steps: (1) Put the guider under the coracoid close to the cortical; (2) drill proximal clavicle; (3) place the titanium cable; (4) perforate distal clavicle, (5) reset the acromioclavicular joint and lock titanium cable; and (6) suture the acromioclavicular ligament. An independent reviewer conducted functional testing of these patients, including the use of coracoclavicular distance (CCD), visual analog scale (VAS) scores, and Constant–Murley scores (CMS).

**Results:**

All patients are presented following at a median duration of 15 months (12-24months) after the surgery. All patients based on X-ray evaluation and clinic evaluation. The median CCD was 7.5 (6–14) mm, the VAS score was 0.55 (0-2), the CMS score was 95.5 (92-99). One patient had subluxation again at the final follow-up based on X-ray examination.

**Conclusions:**

This study demonstrates that the AC joint fixation anatomically with double-bundle titanium, acquired excellent outcomes in terms of the recovery of shoulder joint function and radiographic outcomes. It has a low complication rate and need not remove the hardware.

## Introduction

Acromioclavicular (AC) joint dislocation is a common disease in upper limb trauma, accounting for 2 to 16% of total joint dislocations and 12% of shoulder injuries [1]. When AC joint dislocation occurs, it produces shoulder pain, and movement of the entire upper extremity. AC joint dislocations can be classified according to the Rockwood classification based on the degree and direction of dislocation. Types I or II of AC dislocation are incomplete injury with an intact CC ligament, and treated conservatively; types IV-VI of AC dislocation are severe injury with complete injury with an intact CC ligament need operative treatment; type III injury involves tears of both the AC and CC ligaments, the optimal treatment is controversial, surgical intervention may be appropriate for some patients who was a laborer, elite athlete [2].

Different surgical fixation methods were available for the treatment of AC dislocations. However, no consensus regarding the method has been reached. In general, operative management includes reconstructing the CC ligament or rigid internal fixation of the AC joint [3]. Many factors affect the effects of different technologies, such as the type of injury, method of treatment, and type of reconstruction. The reconstruction technique for the coracoclavicular ligament was developed in recent decades. The ideal technique should involve anatomical reconstruction and double-bundle reconstruction (conical ligament and trapezoid ligament) and provide a stable reduction, use simple fixation methods and minimize complications, especially acromioclavicular joint subluxation or dislocation and of the clavicle [3–5]. There are currently two surgical methods used: The first method uses rigid fixation methods (screws, hook plate); however, these implants have many complications, such as plate or screw breakage, dislocation, and loss of reduction. Moreover, early implant removal is often required due to bone erosion, subacromial impact, and shoulder pain [6–8]. The second method involves reconstruction of the CC ligament (such as with suture buttons, suture anchors, tendon grafts, and synthetic slings). Complications related to the use of these devices include ligament failure, loss of reduction, foreign body reaction, bony erosion, iatrogenic fractures, and suture rupture [9, 10].

As a consequence, we seek for a solid, elastic material to improve treatment outcome with minimally invasive methods [11]. Titanium cable had the above advantages; we invented the guider through titanium cable. In this study, we will use a technique for reduction of the AC joint using double-bundle titanium cables under the guider. The pitfall and perils of the key steps are provided (Table 1).

**Table 1:** Order of steps with pitfalls and pearls

Surgical steps	Pitfalls	Pearls
Open dissection	Incision needs anterior of the clavicle. Deltoid splitting is necessary approach and deltoid damage. Torn intra-articular disk which is not cleared may lead postoperative pain.	A 4-5cm incision allows easy exposure to the AC joint and the clavicle. Detachment of the a little anterior deltoid allows easy access to the upper surface of the coracoid process. Exploration of the AC joint is important to remove the torn intra-articular disk and suture. The AC ligament and AC joint capsule
Guide insertion	It leads injury to the brachial plexus, suprascapular nerves, and blood vessels around the coracoid process if the guard is not corrected.	Coracoid process subperiosteal dissection, the guard is glued to undersurface of the coracoid process and C-arm confirmed.
Clavicular holes	Holes larger than 5 mm may result in a stress fracture of the clavicle. Holes on the same line leads to nonanatomic fixation. Improper sites lead to nonanatomic reconstruction.	A small diameter (≤3 mm) of the hole can avoids clavicular fractures. The clavicular holes: one is proximal hole, slightly posterior on the clavicle which apart 4 cm from the distal end of the clavicle; the other one is distal hole, slightly anterior on the clavicle which apart 2 cm from the distal end of the clavicle.

The purpose of this study was (1) to find an anatomical reconstruction CC ligament method; (2) to make the AC joint reconstruction more precise and stable, so that reduced complications; and (3) to provide evidence and support for clinical extensive application of the novel technique.

## Methods

### Patients

The study performed in our trauma center, from June 2016 to January 2020, approved by the Ethical Committee of the Xiaoshan 1^st^ People’s Hospital of China. Twenty patients with acute high-grade ACJ dislocation (Rockwood types III–V) underwent double-bundle titanium cable reconstruction of the CC ligament. All patients agreed that medical data, including their personal and radiographic photographs.

The inclusion criteria were as follows: (1) more than 18 years old; (2) acute AC joint dislocation (less than 3 weeks after trauma); (3) high-grade injury (Rockwood types IV-VI dislocations, and type III patients with higher requirements), (3) at least 12 months of follow-up.

### Operative technique

**Fig. 1:**
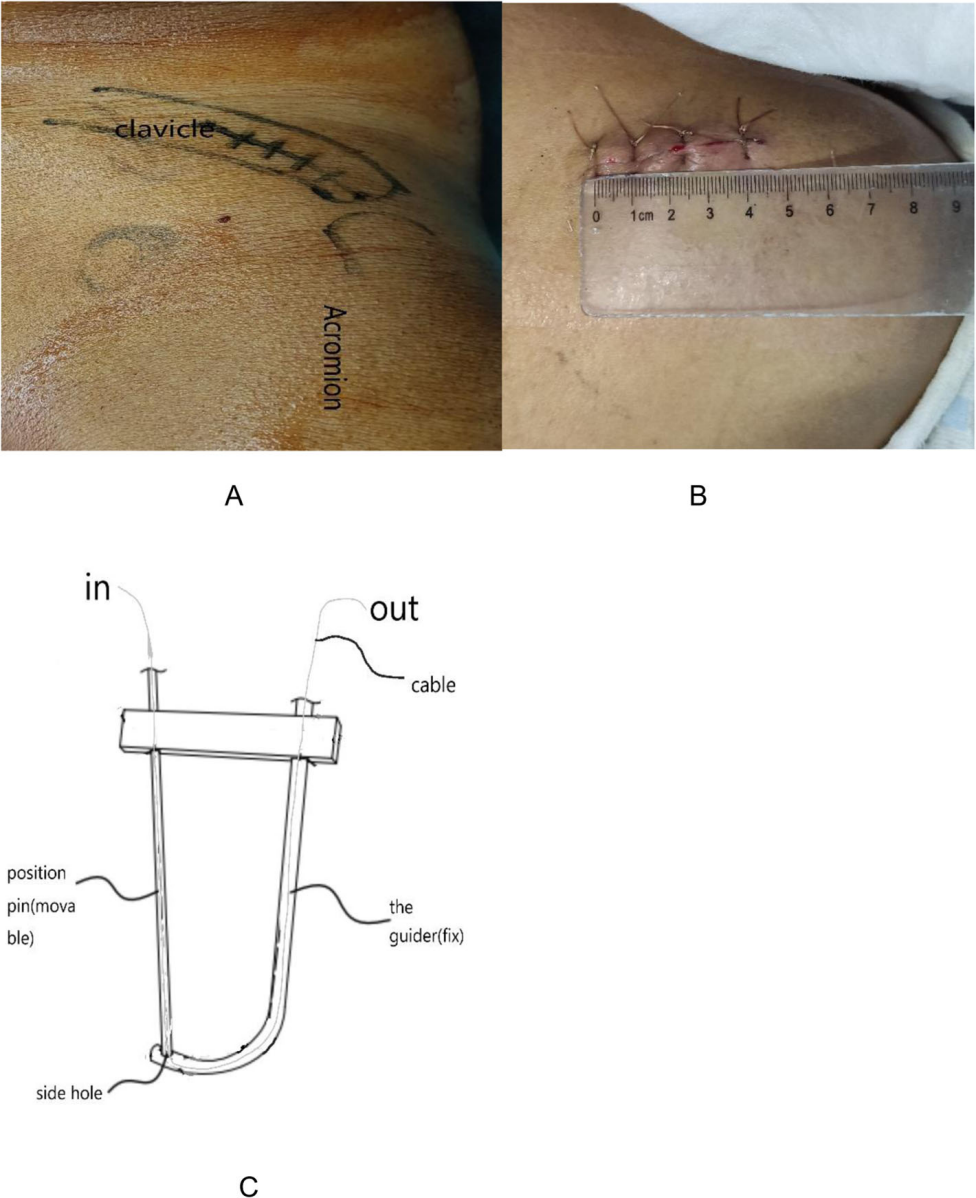
(**a**, **b**) The skin incision is approximately 4-5 cm; (**c**) sketch map of the guider

**Fig. 2:**
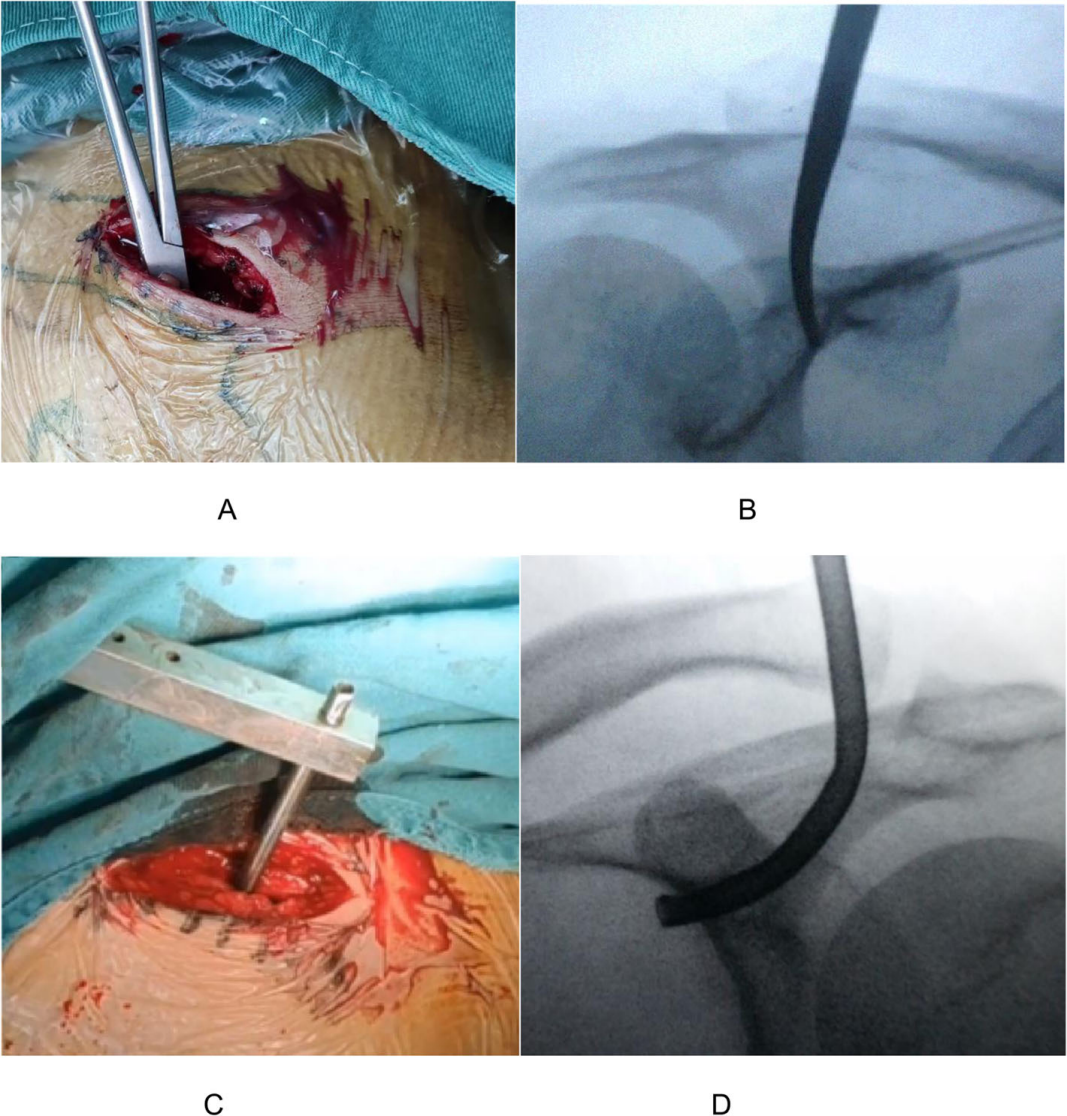
(**a**, **b**) The vascular clamp was inserted along the lateral edge of the base of the coracoid process and confirmed under C-arm guidance, and a tunnel was created; (**c**, **d**) the guider was inserted through the tunnel

**Fig. 3:**
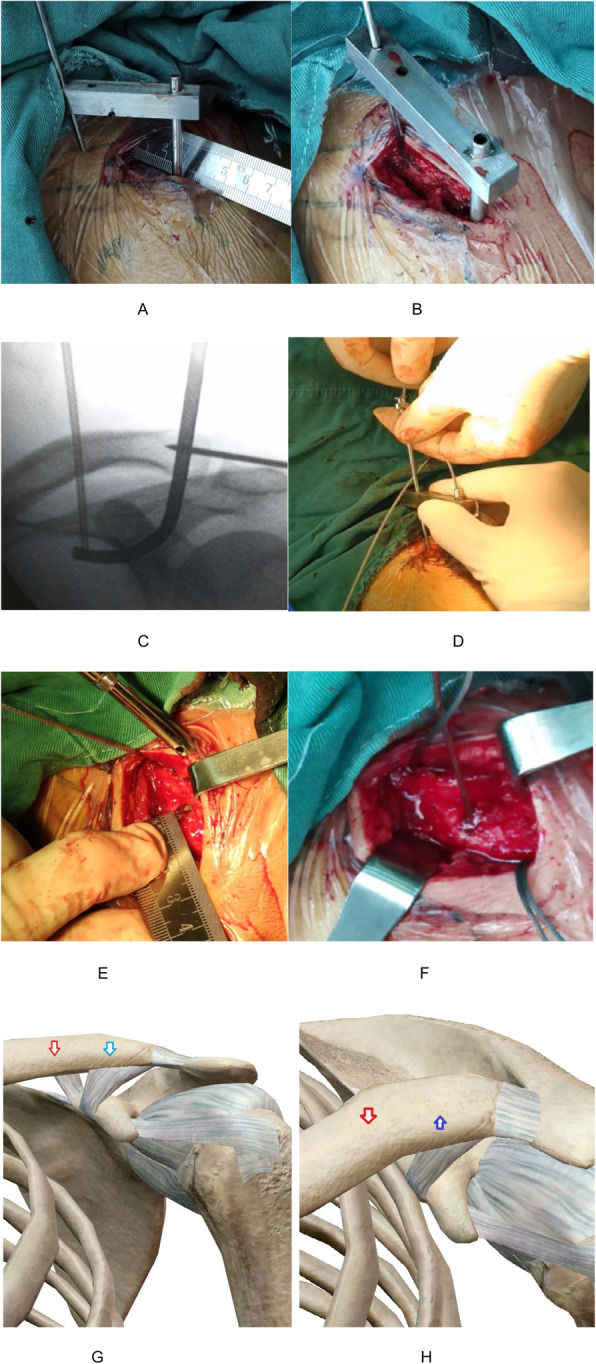
(**a**, **b**, **c**, **d**) The proximal hole was drilled, and a titanium cable was inserted; (**e**, **f**) The distal hole was drilled; (**g**, **h**) sketch map of the proximal hole (red arrow) and the distal hole (blue arrow)

**Fig. 4:**
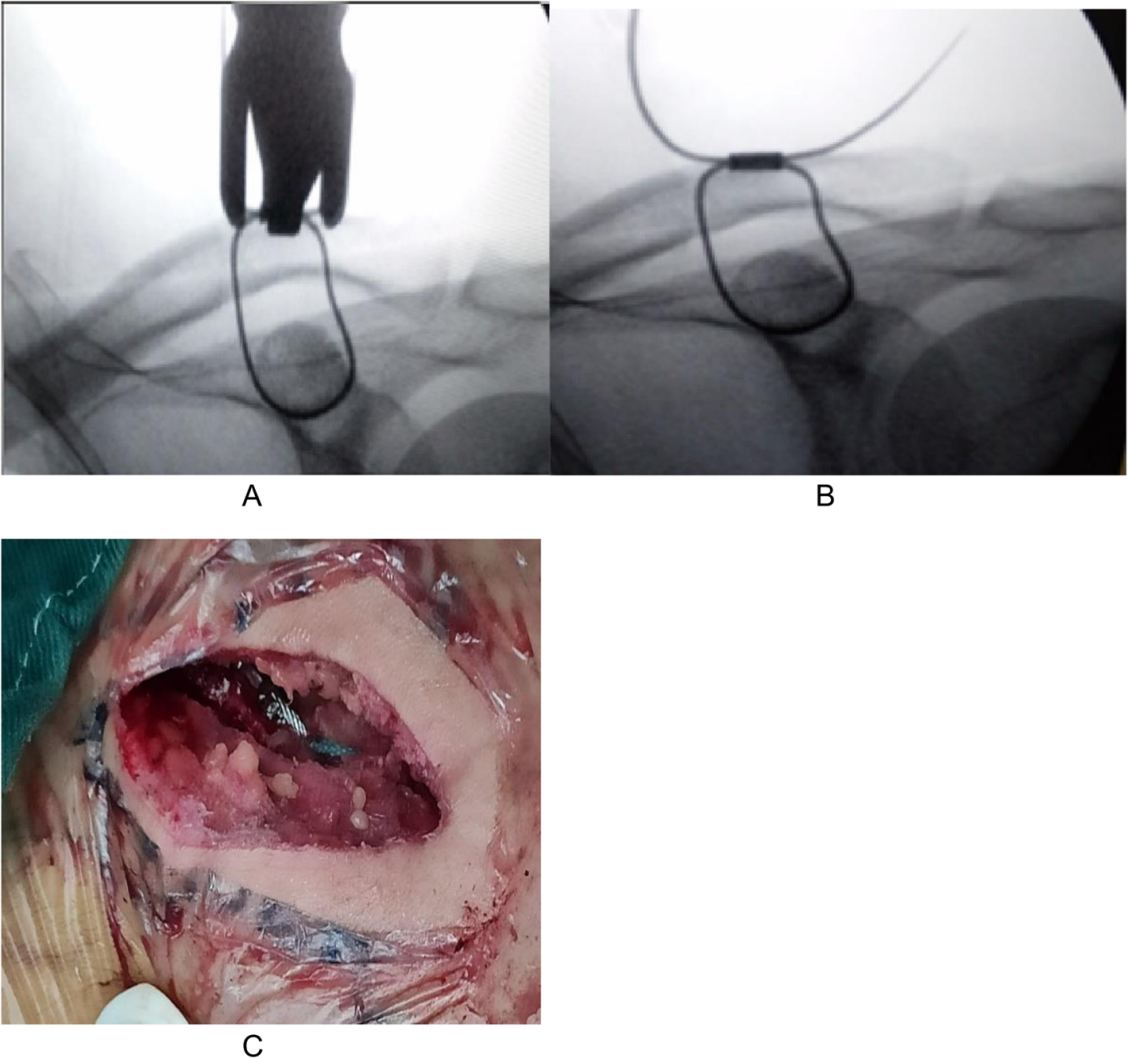
(**a**, **b**) The AC joint was reduced, and the titanium cable was locked; (**c**) the AC ligament and capsule were sutured with no. 5 Ethibond (Johnson & Johnson)

**Fig. 5:**
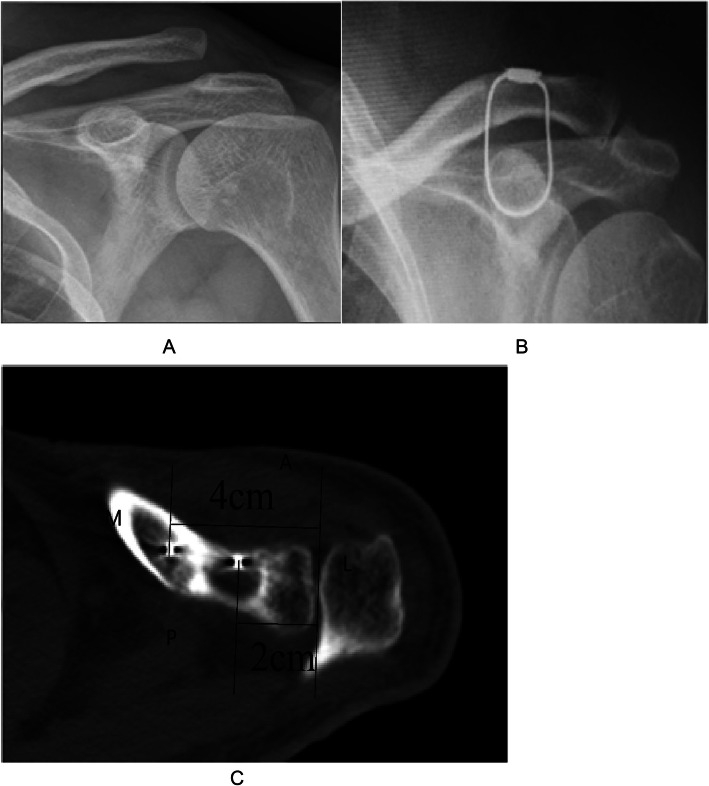
(**a**) Preoperative X-ray of the shoulder; (**b**, **c**) Postoperative X-ray and CT of the shoulder at the final follow-up 12 months after surgery

The operation was performed under general anesthesia. The patient was placed in a beach chair position, his shoulders were padded with a 6-cm-thick cushion, and his head was biased to the healthy side to ensure that the clavicle had sufficient passage and the patient’s arm remained adducted. The surgeon faced the shoulder being operated on and the distal end of the clavicle, and the surface of the coracoid was exposed. An obese person’s acromioclavicular joint should be marked using fluoroscopy. A fluoroscopy unit with a C-arm (to visualize the entirety of the clavicle from anteroposterior and apical oblique angles) was positioned on the contralateral side. A skin incision of 4-5 cm was made at the inner edge of the acromioclavicular joint (Fig. 1a, b). The skin and subcutaneous tissue were incised, and the dislocated acromioclavicular joint was exposed. The free torn tissue of the joint was cleaned, a vascular clamp was inserted along the lateral edge of the base of the coracoid process and confirmed under C-arm fluoroscopy guidance, and a tunnel was created and marked at the outer end of the clavicle (Fig. 2a, b). The guider (Fig. 1C) was inserted into the lower edge of the coracoid process along the tunnel at the clavicle mark, and the guider was confirmed to be closer to the lower edge of the base of the coracoid process with C-arm fluoroscopy (Fig. 2c, d). The side hole of the guider slightly exceeded the inner edge of the coracoid process. The positioning pin (1 mm K-wire) was inserted from the positioning hole of the guider 4 cm posteromedial to the tip of the distal clavicle (the footprint of the conoid ligament) (Fig. 3a, b). After a 2.5-mm hollowed drill was reamed, a 2.5-mm hollowed positioning pin was inserted into the side hole of the guider. After confirmation using C-arm fluoroscopy, the positioning pin and the guider were integrated (Fig. 3c, d). The titanium cable was inserted from the hollowed positioning pin to the guider, and the guider and hollowed positioning pin were removed. A 2.5-mm hole was drilled 2 cm from the distal end of the clavicle (the footprint of the trapezoid ligament) (Fig. 3e, f). Location of proximal hole (the footprint of the conoid ligament) and distal hole (the footprint of the trapezoid ligament) (Fig. 3g, h). The distal titanium cable was inserted into the clavicle, the acromioclavicular joint was reduced, the titanium cable was pressed into the anatomic position of the acromioclavicular joint, and the titanium cable knot was locked (Fig. 4a, b). The AC ligament, capsule, and deltotrapezoid fascia (DTF) were sutured with no. 5 Ethibond (Johnson & Johnson) (Fig. 4c). The torn fascia was sutured, and the skin was closed. A typical case is shown preoperatively (Fig. 5a) and 12 months postoperatively (Fig. 5b, c) in Fig. 5.

### Postoperative rehabilitation

The patient’s arm was fixed with a sling for 6 weeks after surgery. Passive and active-assisted abductions were limited to forward motion to 30° for 2 weeks, 45° for 2 weeks, and 60° for another 2 weeks. Strengthening exercises were implemented after 3 months, and the patients gradually returned to work as early as 6 months after surgery.

### Patients’ evaluation

All patients were followed up at a median duration of 15 months (12-24 months) after the surgery (Table 2). The follow-up evaluations were performed by two doctors, who were not the operating surgeons.

**Table 2:** Patients’ information

No.	Gender	Age	Side	Type	BMI	Follow-up (m)	VAS score		Constant score		Work (m)	Complications
							Pre	Post	Pre	Post		
1	F	47	R	V	20	24	5	1	32	93	8	None
2	M	59	L	V	32	22	3	0	35	97	6	None
3	M	62	L	V	28	19	4	0	36	96	7	None
4	F	58	R	IV	24	12	5	0	28	94	9	None
5	M	39	R	V	25	17	6	0	27	98	8	None
6	M	45	R	IV	35	12	7	1	29	96	8	None
7	F	37	R	V	30	12	4	0	30	94	6	None
8	M	49	L	III	27	17	5	0	36	96	6	None
9	M	46	L	V	25	13	6	0	32	95	6	None
10	M	62	L	IV	31	14	7	1	29	91	0.5	Sublux
11	F	52	R	V	19	12	3	0	38	92	6	None
12	F	26	R	III	24	12	4	0	42	99	6	None
13	M	59	L	IV	32	15	7	0	36	94	7	None
14	M	48	L	IV	26	17	6	1	29	96	7	None
15	M	21	R	V	19	12	6	1	32	92	8	None
16	F	29	R	IV	21	21	4	0	35	91	6	None
17	M	68	R	IV	31	12	5	0	35	94	8	None
18	M	41	R	V	27	16	6	1	32	92	5	None
19	F	59	R	IV	28	15	7	0	28	89	6	None
20	F	62	R	V	32	12	4	1	34	92	7	None
21	M	24	R	III	21	12	5	1	32	91	6	None

Type, Rockwood classification; Pre, pre-operation; Post, post-operation of 1 year later; work, return to work; F, female; M, male; Age, years old; Sub, subluxation

Radiographic assessment include X-ray films were taken 2 days, 3 months, 6 months, and 1 year after the operation to observe the reduction and maintenance of dislocation and the coracoclavicular distance (CCD).

Clinical assessment include operation time, intraoperative hemorrhage, post-operative complications including infection, visual analog scale (VAS) scores, and shoulder function score of Constant-Murley (CMS) were observed and recorded.

### Statistical analysis

Distributions of data were analyzed by SPSS 22 (American SPSS company) software, VAS score, and Constant-Murley score14, and the Mann–Whitney *U* test (for VAS score) and the *t* test (for CCD and CMS) was used; *P* < 0.05 was considered statistically significant.

## Results

The operation time was approximately 42 (35-62) min, and the median blood loss was 85 (60-100) ml. There were no significant complications (no infection or neurovascular injury), one patient (Rockwood type IV) exhibited slight complication: subluxation of the AC joint 1 month postoperatively because of excessive activity. There was no pain or limitation of joint movement of this case.

The median CCD was 18 (13–26) mm at the time of diagnosis, and it was 7.5 (6–14) mm after the operation 1 year later, representing a significant difference (*t* = −20.7 *P*< 0.001). The average VAS score preoperatively was 4.9 (3–7), and it improved to 0.55 (0-2), including the patient with subluxation, the difference was statistically significant (Wilcoxon test, median = −6, *P* < 0.001). The average CMS preoperatively was 32.8 (27-36), and it improved to 95.5 (92-99), the difference was statistically significant (*t* = 60.5, *P* < 0.001).

## Discussion

From the literature [12], they considered Rockwood [13] types IV-VI (include some type III who was a laborer, elite athlete) dislocations to be high-grade dislocations and to require surgery. Surgical procedures focused on anatomic reconstruction of the coraco-clavicular ligaments. Biomechanical studies showed that double coraco-clavicular tunnel technique results in a significant higher stability than single coraco-clavicular tunnel technique [14]. We used optimal clavicular tunnel placements to install a double-bundle titanium cable reconstruct CC ligament (the trapezoid and conoid ligaments) in our study. As noted by Rios and colleagues, the distance from the lateral edge of the clavicle to the medial edge of the conoid tuberosity is approximately 45 mm in males and 40 mm in females, whereas the distance to the center of the trapezoid tuberosity is 25 mm in males and 22 mm in females [15]. We used a 1.3-mm-diameter titanium cable and a guide surrounding the coracoid to reduce and fix the acromioclavicular joint using double-bundle titanium cable fixation distances of approximately 4 cm and 2 cm from the distal end of the clavicle (the footprint of the CC ligament). This technique restores the anatomy and biomechanical properties of the native ligaments. Double-bundle titanium cable fixation increases the vertical and horizontal stability of the acromioclavicular joint, making the acromioclavicular joint in close contact with a certain amount of micromotion, consistent with the normal biomechanics of the acromioclavicular joint. The CC ligament fixation method with two small-width holes (2.5 mm) can predictably reduce the risk of fracture of the clavicle, and the coracoid process has no hole, so it has no risk of fracture. In addition, drilling small holes result in less damage to pre-existing torn CC ligaments.

We believe that an injury of less than 3 weeks is an acute injury. Therefore, this procedure is only applicable to patients who have undergone repair of acute acromioclavicular joint dislocation within 3 weeks after injury. Most experts agreed that the damaged coracoclavicular ligament can repair itself in an acute injury. The unique feature of our technique is the use of a titanium cable to reduce the acromioclavicular joint without coronoid process exposure, which avoids neurovascular damage around the coracoid and involves less damage to the torn coracoclavicular ligament than other techniques. The coracoclavicular ligament can be repaired with the normal CC interval distance consistently because of the double-bundle titanium cable implant. After a mean follow-up of 15 months, we did not record any tunnel fracture or implant failure. The patients revealed excellent radiological and clinical results, except one subluxation of the acromioclavicular joint. This one goes to work 2 weeks after operation. In contrast to the present study, reconstruction of the coracoclavicular ligament also includes an Endobutton system, adjustable-loop-length suspensory fixation, suture anchors, an artificial tendon, absorbable sutures, and autogenous tendon transplantation [16, 17]. Among them, the Endobutton system is the most widely used because it can provide a higher fixed strength. This technique involves single-bundle fixation with a swing effect of the clavicle. The single application cannot provide and maintain strength equivalent to that of the CC ligament, and the incidence of subluxation of the acromioclavicular joint is higher for Endobutton systems [18, 19]. Because of its swing effect, it may lead to delayed or non-healing of the CC ligament and enlarge the hole diameter, causing clavicular fracture.

Xue et al. [20] found that double Endobutton fixation provides comparable or even higher strength to the intact ligament because it needs to expose the coracoid process, exacerbating surrounding soft tissue damage, including possible neurological and vascular damage, causing shoulder discomfort, extending recovery time and the return to work time. Additionally, the suspension system provides sufficient vertical stability but does not control horizontal displacement.

Cai L et al. [4] found that compared with the single TightRope technique, arthroscopically assisted double TightRope fixation combined with percutaneous acromioclavicular joint cerclage significantly reduced the incidence of horizontal displacement. However, Tae Kang Lim [21] reported that the frequency of operative complications was very high in 61% (11/18) of CC ligament reconstructions with TightRope following arthroscopy. Martetschlager [22] et al. reported that there were 16 cases of complications among 59 cases of CC ligament reconstruction with TightRope under arthroscopy, including three cases of coracoid process and clavicle fracture. Some scholars compared TightRope technology with Kirschner wire, clavicular hook plate, and Bosworth screw technology and found that TightRope technology has similar clinical effects to the other three technologies and did not significantly improve shoulder function. On the other hand, the difficulty of the surgical technique limits its extensive development because of the steep learning curve. Compared with arthroscopic surgery, our method is minimally invasive, similar to arthroscopic surgery, without the steep learning curve of shoulder arthroscopy and equipment requirements. It can be widely carried out in every hospital.

Another major treatment for acute acromioclavicular joint dislocation is the clavicular hook plate, which is still widely used clinically. In the 1970s, clavicular hook plates were used to treat acromioclavicular joint dislocation. The surgical procedure is to first insert the hook end into the shoulder and lift the external scapula and then to fix the plate to the distal end of the clavicle to restore the acromioclavicular joint. Compared with Kirschner wire and Bosworth screw fixation, clavicular hook plate fixation allows the clavicle and scapula to move slightly, facilitating functional exercise early in the postoperative period. The operation is simple, the curative effect is reliable, and it is still the mainstream for treating acromioclavicular joint dislocation in primary hospitals [23, 24]. However, clavicular hook plate fixation easily causes subacromial impact, subacromial osteolysis, shoulder fracture, clavicular hook prolapses and bursitis, distal clavicle bone atrophy, and other complications and requires surgical removal of the plate. The shoulder joint will remain in a restricted state until the hardware is removed [25, 26].

This technique reduces and fixes the AC joint (anatomical reconstruction of the CC ligament) without the need to remove the implant. In all of our cases, a double-bundle titanium cable was used. After 15 months of follow-up, we did not find clavicle or coracoid process fracture or implant failure. Clinical outcomes revealed that all patients had good function except one patient who developed subluxations of the acromioclavicular joint. The advantages and limitations of the technique are provided (Table 3).

**Table 3:** Advantages and limitations of the technique

**Advantages**	
Double-bundle titanium cable is approximately at the coracoclavicular ligament footprint on the clavicle. Double-bundle titanium cable fixation is simulated the trapeze ligament and the pyramidal ligament, relax the coracoclavicular ligament and allow it to be repaired. Anatomic AC reduction is provided. The guide passes across the coracoid process without the coracoid process being exposed. A good acromioclavicular joint reduction Early reconstruction is better than late reconstruction. A more stable reduction is provided with less failure than repair. The two holes are drilled at anatomic sites of the native coracoclavicular ligament attachments (2cm, 4cm). The holes are less than 3 mm to avoid fracture of the clavicle. The technique is simple and inexpensive. The technique avoids secondary surgery.	
**Limitations**	
Performed for acute cases, we have no experience with chronic cases. For BMI>30 patients, early weight-bearing shoulder motion is not suggested. Coracoid fracture is a contraindication. Bone tunnel enlargement and reduction loss The sample size is not large enough in general. The current study is prospective, not in a RCT. Need larger sample controlled trials	

In the future, we can use biological composite material instead of titanium cable to reconstruct coracoclavicular ligament, which is more in line with the biomechanics of coracoclavicular ligament [27].

## Conclusions

We prefer titanium cable because it has ductility, elasticity, firmness, and good histocompatibility. A double-bundle titanium cable anatomical reconstruction CC ligament according to the footprints of ligaments is effective procedures for the surgical treatment of AC joint acute dislocations of Rockwood III, IV, and V. Complications are low.

## Data Availability

None of the raw data has been made available in any public repository. The original reports, imaging studies, and outpatient clinic records are retained as per normal procedure within the medical records of our institution.

## Abbreviations

AC: Acromioclavicular
CC: Coracoclavicular
CMS: Constant–Murley score
CCD: Coracoclavicular distance
VAS: Visual analog scale
